# Drug Loaded Gingival Mesenchymal Stromal Cells (GinPa-MSCs) Inhibit *In Vitro* Proliferation of Oral Squamous Cell Carcinoma

**DOI:** 10.1038/s41598-017-09175-4

**Published:** 2017-08-24

**Authors:** Valentina Coccè, Davide Farronato, Anna Teresa Brini, Carla Masia, Aldo Bruno Giannì, Giovanna Piovani, Francesca Sisto, Giulio Alessandri, Francesca Angiero, Augusto Pessina

**Affiliations:** 1 StaMeTec, Department of Biomedical, Surgical and Dental Sciences, University of Milan, Via Pascal 36, 20133 Milan, Italy; 2 Department of Medicine and Surgery, Dental School, University of Insubria, Varese, Italy; 3 I.R.C.C.S. Istituto Ortopedico Galeazzi, Via Riccardo Galeazzi 4, 20161 Milan, Italy; 4 Fondazione IRCCS Cà Granda Ospedale Maggiore Policlinico, Maxillofacial and Dental Unit, Via Commenda 10, 20122 Milan, Italy; 5 Biology and Genetics Division, Department of Molecular and Translational Medicine, University of Brescia, Brescia, Italy; 6 Cellular Neurobiology Laboratory, Department of Cerebrovascular Diseases, IRCCS Neurological Institute C. Besta, Via Celoria 11, 20133 Milan, Italy; 7 Department of Medical Sciences and Diagnostic Integrated, S. Martino Hospital, University of Genoa, Largo R. Benzi 10, 16132 Genova, Italy

## Abstract

Human mesenchymal stromal cells (MSCs) have been widely investigated both for regenerative medicine and their antinflammatory/immunomodulatory capacity. However, their ability to home pathological tissues suggested the development of strategies for using MSCs as carrier to deliver drug into tumor microenvironment. MSCs obtained from different tissues can be loaded *in vitro* with anti-cancer drugs by a simple procedures. In this report, we studied MSCs isolated and expanded from gingival papilla (GinPa-MSCs), by testing their ability to uptake and release three important anti-neoplastic drugs: Paclitaxel (PTX), Doxorubicin (DXR) and Gemcitabine (GCB). The efficacy of drugs releasing GinPa-MSCs was studied on a pancreatic cancer cell line and confirmed *in vitro* against a line of tongue squamous cell carcinoma (SCC154). Our results demonstrated that GinPa-MSCs efficiently incorporate the drugs and then released them in active form and in sufficient amount to produce a dramatic inhibition of squamous cell carcinoma growth *in vitro*. If compared with other MSCs sources, the collection of GinPa-MSCs is poorly invasive and cells can be easily expanded and efficiently loaded with anti cancer drugs. In particular, gemcitabine loaded GinPa-MSCs provide a good “cell-mediated drug delivery system” for a future potential application in the context of the oral oncology.

## Introduction

Head and neck carcinoma are primarily originated from lip-oral cavity, pharynx and larynx and 95% of them are classified as squamous cell carcinoma (SCC) These type of cancers are still a significant cause of death in the world^1^, although progress have been obtained both by surgical and chemo-radiation treatment. In fact, these treatments of SCC reduce important side effects, but unfortunately, they are not balanced by significant increase in the survival rate. Treatment failure often results in a tumour recurrence and also metastasis^2^ that must be treated with chemotherapy^3, 4^. In this context, it could be an important goal to develop new approaches both for the first line treatment and in the long run for the second line one. Methotrexate remains a standard for treating patients with advanced, recurrent or metastatic SCC, but also cisplatin/fluorouracil or cisplatin/taxane as well as single-agent taxane are considered alternatives^5^. In a recent Phase II clinical trial, the gemcitabine-paclitaxel treatment has been also considered as second line treatment applied to patients who did not respond to cisplatin in association with radiotherapy^6^.

To develop more specific and less toxic treatments of cancer, the use of cells, mesenchymal stromal cells (MSCs) in particular, has been proposed for the drug delivery. In fact, MSCs seem to have the capacity to migrate towards inflammatory microenvironments and to reach tumour sites after their systemic administration. To develop new therapeutic approaches based on anti-cancer molecules MSC-mediated delivery, engineered MSCs^7, 8^ were used; in addition, the ability of MSCs to uptake and release drugs without any genetic manipulation^9–11^ was also exploited. Among the different sources of MSCs, studies have been conducted using bone marrow MSCs that have some limitations particularly concerning the discomfort caused in the donors. For this purpose, the ability of different sources of MSCs to uptake/release drugs^9–13^ has been evaluated. Among the different MSC sources, those from gingival could be an interesting model as they are easy to obtain with minimal invasive procedure and easy to expand with a few passages. In this work, we studied their ability to uptake the anti-neoplastic drugs Doxorubicin (DXR) and Gemcitabine (GCB) in comparison with Paclitaxel (PTX). We put particular attention in verifying the *in vitro* anticancer activity of the drug-releasing GinPa-MSCs against a tongue squamous cell carcinoma cell line (SCC154).

## Methods

### Ethics statements

Samples from adult donors were collected after written informed consent in accordance with the Declaration of Helsinki; the approval for sample use was obtained from the Institutional Ethical Committee of IRCCS Cà Granda Ospedale Maggiore Policlinico of Milan (#978).

### Mesenchymal stromal cells

Human gingival MSCs were isolated, expanded and characterized as previously described^13^. Briefly, the discarded gingival tissue from reductive gingivoplasty were collected in sterile conditions and transferred to the Cell Culture Laboratory to be processed. The samples were minced with surgical scissors, digested by type I Collagenase (50 U/ml, Life technologies, UK) at 37 °C under stirring condition for 3 hours, and centrifuged (300 × g for 10 minutes). Cellular pellets were plated in 25 cm^2^ flask in Dulbecco’s modified Eagle’s medium with high glucose (DMEM HG) supplemented with 10% Foetal bovine serum (FBS) and 1% L-glutamine (all reagents Euroclone, UK), maintained at 37 °C, 5% CO_2_, and selected by plastic adherences. Primary cultures were analysed for their proliferation rate (Population doubling time), clonogenicity (CFU-F assay), expression of the typical mesenchymal stem cell markers (high expression of CD73, CD90 and CD105; a mild expression of CD14 and negative for CD45) and multi-differentiative ability towards mesodermal lineages (osteogenic, adipogenic and chondrogenic differentiation).

### Tumor cell line

The human squamous cell carcinoma line (SCC154)^14^ and the human pancreatic adenocarcinoma cell line CFPAC-1^15, 16^ were provided by Centro Substrati Cellulari, ISZLER (Brescia, Italy). Cells were maintained in DMEM HG +10% FBS +1% Non essential aminoacid (SCC154) and Iscove modified Dulbecco’s medium (IMDM) +10% FBS (CFPAC-1), by 1:5 weekly dilution. All reagents were provided by Euroclone, UK.

### Drug sensitivity of GinPa-MSCs, SCC154 and CFPAC-1 to PTX, DXR and GCB

The sensitivity of GinPa-MSCs to chemotherapeutic drugs Paclitaxel (PTX, kindly provided by Freseneius-Kabi. Italy; stock solution 6 mg/ml), Doxorubicin hydrochloride (DXR, Alexis biochemicals, Switzerland, stock solution 5 mg/ml) and Gemcitabine hydrochloride (GCB, Accord Healthcare Limited, UK, stock solution 38 mg/ml), was determined by adding the drugs at increasing concentrations from 0.01 to 10 μg/ml (cytotoxicity assay) or from 0.39 to 100 ng/ml (anti-proliferative assay). The MTT (3-(4,5-dimethyl-2-thiazolyl)-2,5-diphenyl-2-Htetrazoliumbromide) assay was performed after 24 hours (cytotoxicity) or after 7 days (anti-proliferation)^17^. The inhibitory concentrations (IC_50_ and IC_90_) were determined according to the Reed and Muench formula^18^.

### Drug loading of GinPa-MSCs with PTX, DXR and GCB

The drug loading of GinPa-MSCs was performed by priming the cells according to a standardized procedure that use high drug dosage as previously described^9, 13^. Briefly, subconfluent cultures (2 × 10^4^ cells/cm^2^) were exposed to 2 µg/ml PTX, or DXR, or GCB for 24 hours. Then, the cells were washed twice with PBS, trypsinized and washed twice in Hank’s solution (HBSS, Euroclone UK). Drug-primed cells (GinPa-MSCs/PTX, GinPa-MSCs/DXR, GinPa-MSCs/GCB) were then seeded in a 25 cm^2^ flask in DMEM high glucose with 10% FBS and 2 mM L-glutamine (Euroclone, UK) to release the drug. After 48 hours, conditioned media (CM), (GinPa-MSCs/PTX CM, GinPa-MSCs/DXR CM GinPa-MSCs/GCB CM) were collected and tested *in vitro* for their anti-proliferative activity on SCC154 cells and CFPAC-1 cells (used as standard assay). Conditioned Media from unprimed MSCs were used as control. To determine the amount of drug internalized and not released, cells collected after the releasing phase were lysed by three sonication cycles of 0.4 second pulse cycle and 30% amplitude each (Labsonic U Braun). Lysates from PTX, DXR and GCB primed cells (GinPa-MSCs/PTX LYS, GinPa-MSCs/DXR LYS, GinPa-MSCs/GCB LYS) and unprimed GinPa-MSCs were tested for their anti-proliferative activity.

### *In vitro* anti cancer assay on SCC154

The inhibitory effect of CM from drug loaded GinPa-MSCs and cell lysates were evaluated on SCC154 proliferation by the MTT assay^17^ and the inhibitory concentration IC_50_ was determined according to the Reed and Muench formula^18^. The anti-tumoral activity of GinPa-MSCs/PTX CM, DXR CM and GCB CM were compared to that of the pure drugs (PTX, DXR and GCB) alone and expressed as drug equivalent concentration (DEC), according to the following algorithm: DEC (ng/ml) = IC_50_ Drug(ng/ml) × 100/ IC_50_ CM (µl/well). The IC_50 Drug_ is the concentration (ng/ml) of pure drug able to inhibit SCC154 proliferation by 50%; IC_50_CM is the volume (µl/well) of CM inhibiting SCC154 proliferation by 50%. To evaluate the drug released by a single primed cell (CDR = cell drug release), an arbitrary internal parameter based on the ratio between the DEC and the number of seeded cells [CDR (pg/cell) = DEC (ng/ml) × 10^3^ × CM volume (ml)/number of seeded cells] was used.

The effect of drug released by GinPa-MSCs to SCC154 was also observed as damage exerted on tumor cells plated in 96 well plates (20000 cells/well). After 24 hour incubation at 37 °C and 5% CO_2_ to allow cellular adhesion, the culture medium was replaced by GinPa-MSCs lysate from both unprimed and PTX, DXR or GCB-primed cells. After 48 hour incubation, cells were visualized with optical microscope to evaluate the apoptotic and necrotic effects.

### Secretome analysis

The CM from GinPa-MSCs were analysed for their cytokines/chemokines content by using multiplex bead-based assays on xMAP technology (Bio-Plex Human Cytokine 27-Plex Panel; Bio-Plex Human Group II Cytokine 21-Plex Panel; Biorad Laboratories). As the assay is forecasted to analyse 48 different cytokines, in our study we considered only the cytokines that were found in the conditioned media at levels >of 1,500 pg/ml.

### Expression of Concentrative Nucleoside Transporter-1 (hCNT-1) and equilibrative nucleoside transporter 1 (hENT-1) by GinPa-MSCs

The expression of hCNT-1and hENT-1 gene was evaluated by qRT-PCR technique as previously described^19, 20^. Briefly, RNA was isolated from cultured cells by Trizol (Life technologies, USA) and transcribed into cDNA using random primers, ribonuclease inhibitor and M-MLV reverse transcriptase (Life technologies, USA). The qRT-PCR analysis was performed using SYBR Green on a real-time PCR system 7500 and 7500 System SDS Software (Applied Biosystems, USA).

### Statistical analysis

Data are expressed as average ± standard deviation (SD). Differences between mean values were evaluated according to Student’s t-test performed by GRAPHPADINSTAT program (GraphPad Software Inc., San Diego, CA, USA). p values ≤ 0.05 were considered statistically significant.

The linearity of response and the correlation were studied using regression analysis, by Excel 2013 software (Microsoft, Inc.).

### Data availability

All data generated or analysed during this study are included in this published article.

## Results

### Sensitivity of CFPAC-1 and SCC154 to the anti-proliferative activity of PTX, GCB and DXR

**Figure 1:**
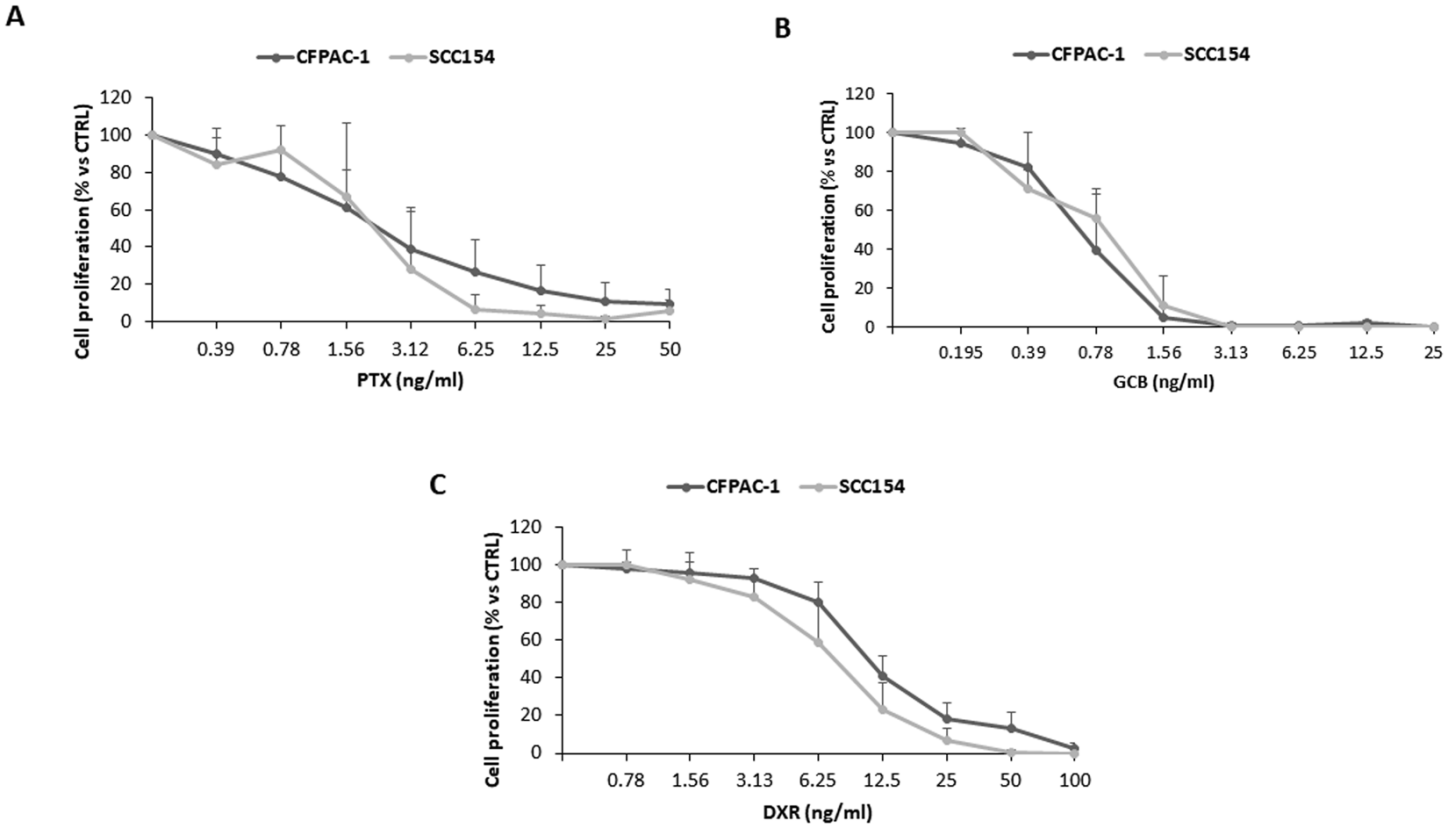
Sensitivity of CFPAC-1 (pancreatic adenocarcinoma line) and SCC154 (squamous cell carcinoma line) to the anti-proliferative activity of PTX, GCB and DXR. The effect of increasing concentrations (from 0.195 ng/ml to 100 ng/ml) of PTX (**A**), GCB (**B**) and DXR (**C**), was evaluated by a 7-day anti-proliferation assay. The effect is expressed as percentage of the Optical Density measured in cultures that did not receive drugs (100% proliferation). PTX = Paclitaxel; GCB = Gemcitabine; DXR = Doxorubicine.

The *in vitro* activity of PTX, GCB and DXR evaluated on the two human carcinoma cell lines CFPAC-1 and SCC154 showed a similar dose-response kinetics (Fig. 1). The CFPAC-1 and SCC154 resulted about 3–4 time more sensitive to GCB (IC_50_ value: 0.66 ± 0.23 ng/ml for CFPAC-1 and 0.95 ± 0.40 ng/ml for SCC154) than to PTX (IC_50_ value: 2.21 ± 1.87 ng/ml for CFPAC-1 and 2.11 ± 1.10 ng/ml for SCC154) and almost 10 times more sensitive if compared to DXR (IC_50_ value: 10.62 ± 2.09 ng/ml for CFPAC-1 and 7.39 ± 2.17 ng/ml for SCC154).

### Sensitivity of GinPa-MSCs to PTX, GCB and DXR

**Figure 2:**
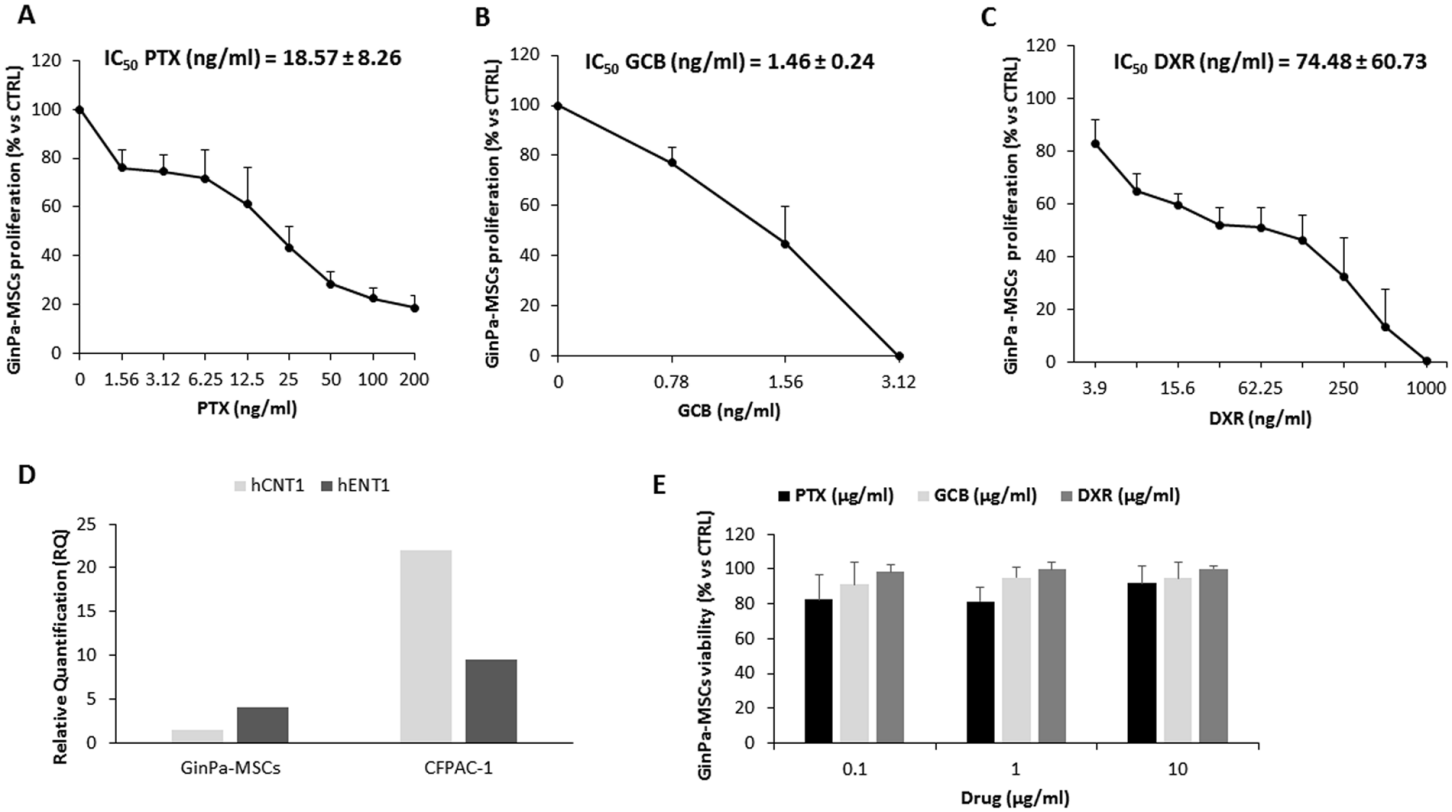
Sensitivity of GinPa-MSCs to PTX, GBC and DXR and hCNT1, hENT1 expression. The effect of increasing concentrations (from 0.78 ng/ml to 1000 ng/ml) of PTX (**A**), GCB (**B**) and DXR (**C**), was evaluated by a 7-day anti-proliferation assay. The effect is expressed as percentage of the Optical Density measured in cultures that did not receive drugs (100% proliferation) and the IC_50_ values by Reed and Muench formula are reported in the graphs. The histogram (**D**) reports the expression of hCNT1 and hENT1 in GinPa-MSCs compared with CFPAC-1 (positive control) evaluated by qRT-PCR analysis as described in Materials and Methods. The histograms reports the mean value of relative quantification (RQ) versus housekeeping gene (GAPDH) performed in duplicate. The cytotoxic activity of PTX, GCB and DXR (**E**) was evaluated at 24 hours of treatment with increasing concentrations of drugs (from 0.1 to 10 μg/ml). The data are expressed as mean ± standard deviation (SD) of cell viability (% of control cells) of three independents experiments.

The three drugs tested for their antiproliferative activity on GinPa-MSCs confirmed that GinPa-MSCs were less sensitive to these anticancer molecules than cancer cells with a range from 2 times for GCB to 51 times for DXR. By comparing the IC_50_ values, GCB resulted the most active molecule (IC_50_ = 1.46 ± 0.24 ng/ml), followed by PTX (IC_50_ = 18.57 ± 8.26 ng/ml) and by DXR (IC_50_ = 74.78 ± 60.73 ng/ml) (Fig. 2A,B,C).

The expression of the two main GCB transporters (hCNT-1 and hENT-1) by GinPa-MSCs was evaluated by RT-PCR compared with that of pancreatic cancer CFPAC-1 cell line (Fig. 2D). The high sensitivity to GCB well correlates with the expression of the main GCB transporter hENT1 that is considered the main carrier involved in gemcitabine transport^21^.

The drug sensitivity of GinPa-MSCs, measured as cytocidal effect after 24 hours of treatment confirmed some degree of resistance. At concentrations up to 10,000 ng/ml GinPa-MSCs, cell mortality of the treated cells was close to 20%. (Fig. 2E). This allowed to apply the standard procedure for drug loading based on high drug dosage treatment (2,000 ng/ml).

### PTX, GCB and DXR uptake-release by GinPa-MSCs assessment on CFPAC-1 cells

**Figure 3:**
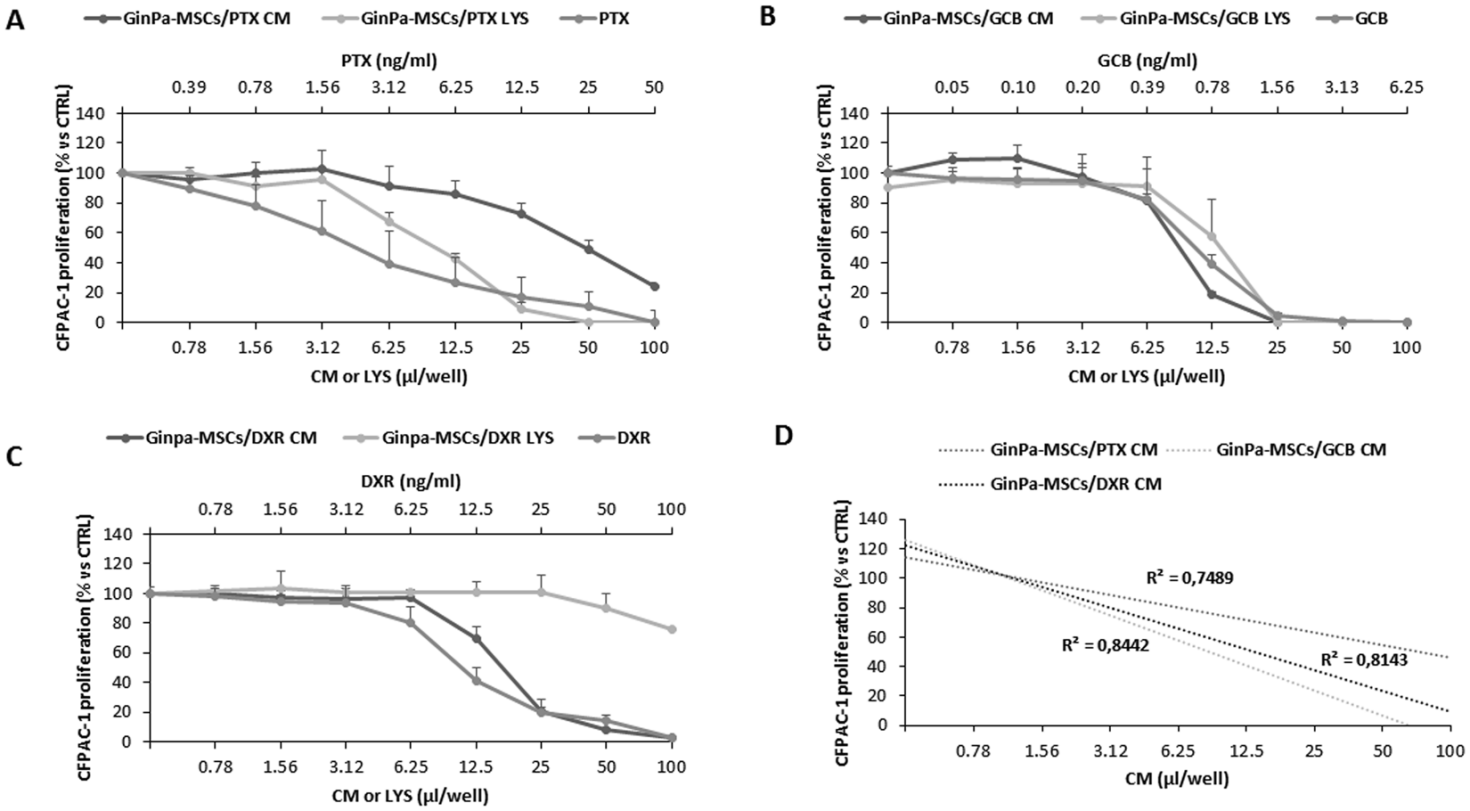
PTX, GCB and DXR uptake and release by GinPa-MSCs tested on CFPAC-1 cells. The effect of conditioned media (CM) and lysate (LYS) from GinPa-MSCs/PTX (**A**), GinPa-MSCs/GCB (**B**) and GinPa-MSCs/DXR (**C**), are expressed as percentage of CFPAC-1 proliferation referred to that of untreated cells (100% proliferation). Values are expressed as mean ± standard deviation (SD) of three independent experiments. The regression and the correlation coefficient (R^2^) of the dose response kinetics are reported in figure (**D**).

**Figure 4:**
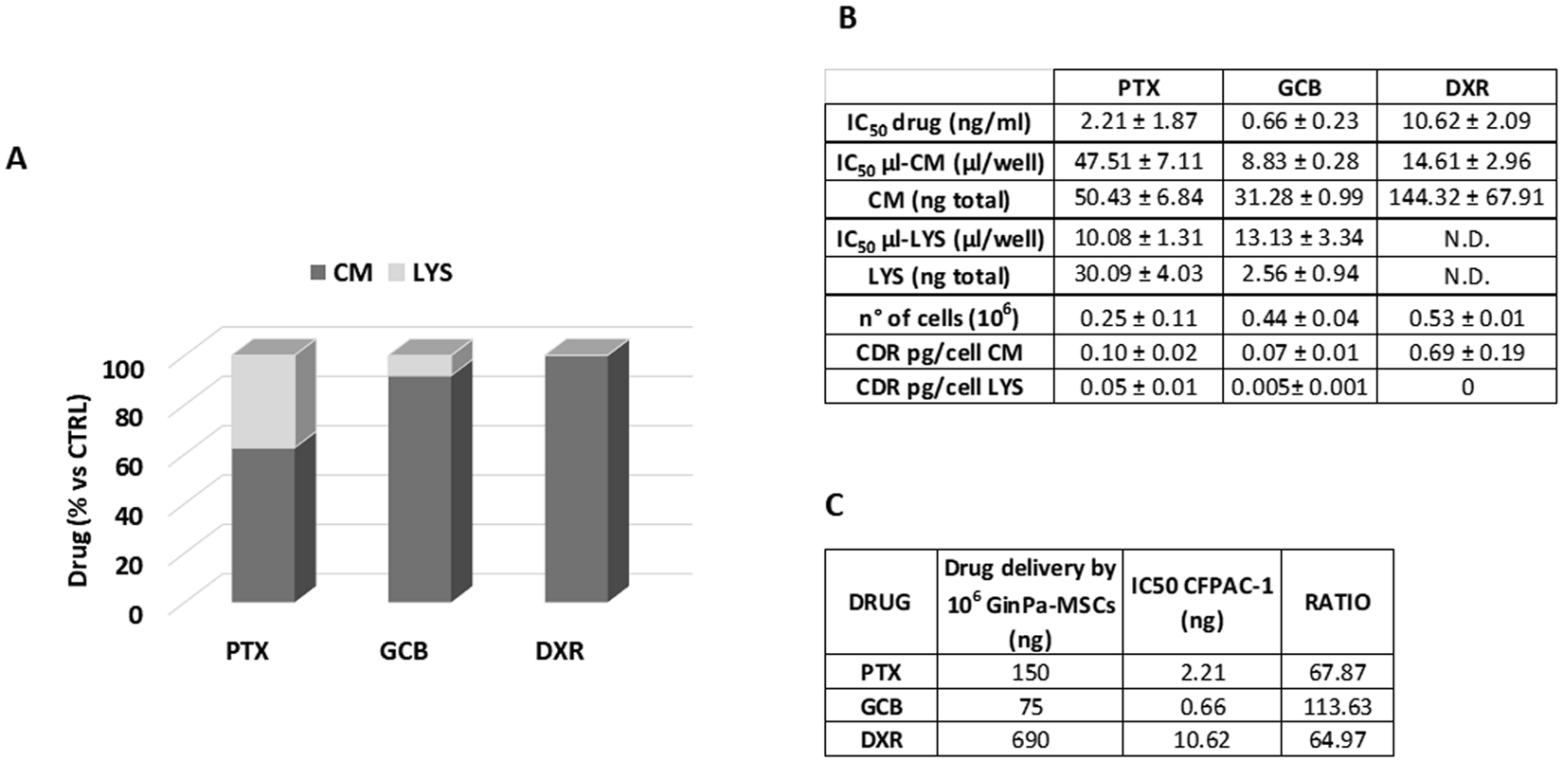
Percentage of drug incorporated and released. The histogram (**A**) reports the percentage of drugs estimated by biological dosage in the conditioned medium (CM) and cell lysate (LYS). The box (**B**) reports the IC_50_ value of the drugs (ng/ml) of the conditioned medium and lysate (µl/well) used to estimate the amount of drugs incorporated (LYS, ng total) or released (CM, ng total) by GinPa-MSCs. Values are expressed as mean ± standard deviation (SD) of three independent experiments. The box (**C**) reports the amount of drugs potentially delivered by 10^6^ GinPa-MSCs and their ratio to the IC_50_ values of each drug. CDR = Single cell drug release.

The conditioned media (GinPa-MSCs/PTX CM, GinPa-MSCs/GCB CM and GinPa-MSCs/DXR CM) and the corresponding cell lysate (GinPa-MSCs/PTX LYS, GinPa-MSCs/GCB LYS and GinPa-MSCs/DXR LYS) from drug-primed cells were tested on the standard laboratory cell line CFPAC-1 (Fig. 3). All the CMs affected CFPAC-1 cell growth according to a dose-dependent, anti-proliferative effect equivalent to the one produced by free PTX (from 0.78 to 100 ng/ml). The cell lysates of PTX and GCB-loaded cells (GinPa-MSCs/PTX LYS, GinPa-MSCs/GCB LYS) exerted a significant inhibition of cell proliferation, while no inhibition was exerted by GinPa-MSCs/DXR LYS that only produced a small, not significant effect at the highest concentration. The above described inhibitory kinetics analyzed by linear regression showed high coefficients of determination (R^2^) ranging from 0.74 to 0.84. The biological dosage of the CM and LYS, based on the standard dose-response regression of the pure drugs enabled the estimation of an equivalent drug concentration (DEC) of incorporated (LYS) and released (CM) drug by a single cell (CDR) ranging from 0.075 pg/cell for GCB to 0.69 pg/cell for DXR (Fig. 4B). The percentage of drug released by cells in the CM referred to the total amount incorporated confirmed that at 24 hour culture, the cells released 62.6% of PTX, 91.8% of GCB and 100% of DXR (Fig. 4A). The drug concentrations released by 10^6^ GinPa-MSCs were 75 ng for GCB, 150 ng for PTX and 669 ng for DXR, respectively, that correspond to values of 67.87, 113.63 and 64.97 times the IC_50_ values, respectively.

### Anticancer activity against squamous carcinoma SCC154

**Figure 5:**
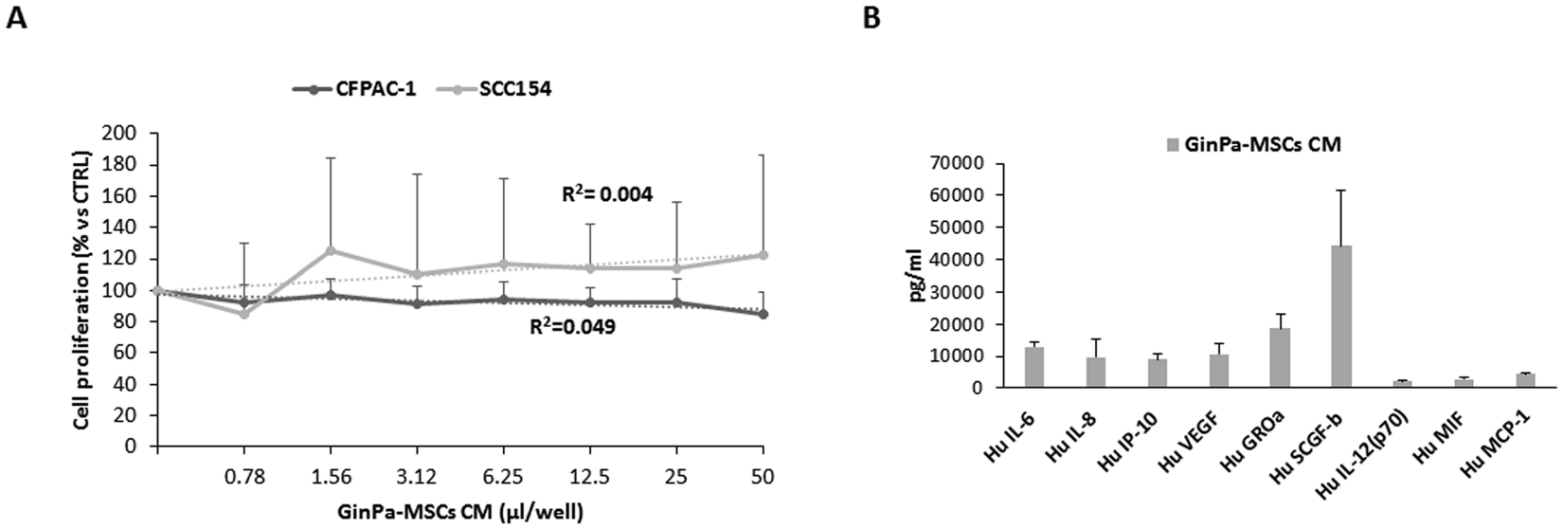
Effect of GinPa-MSCs secretome on proliferation of SCC154. The effect of the physiological conditioned media (CM) from GinPa-MSCs were evaluated as percentage of the proliferation of squamous cell carcinoma (SCC154) referred to the control. The study of the regression (R^2^) is reported. The histogram (**B**) report the amount of the main cytokines detected in the GinPa-MSCs secretoma (only that were present at levels >1,500 pg/ml).

**Figure 6:**
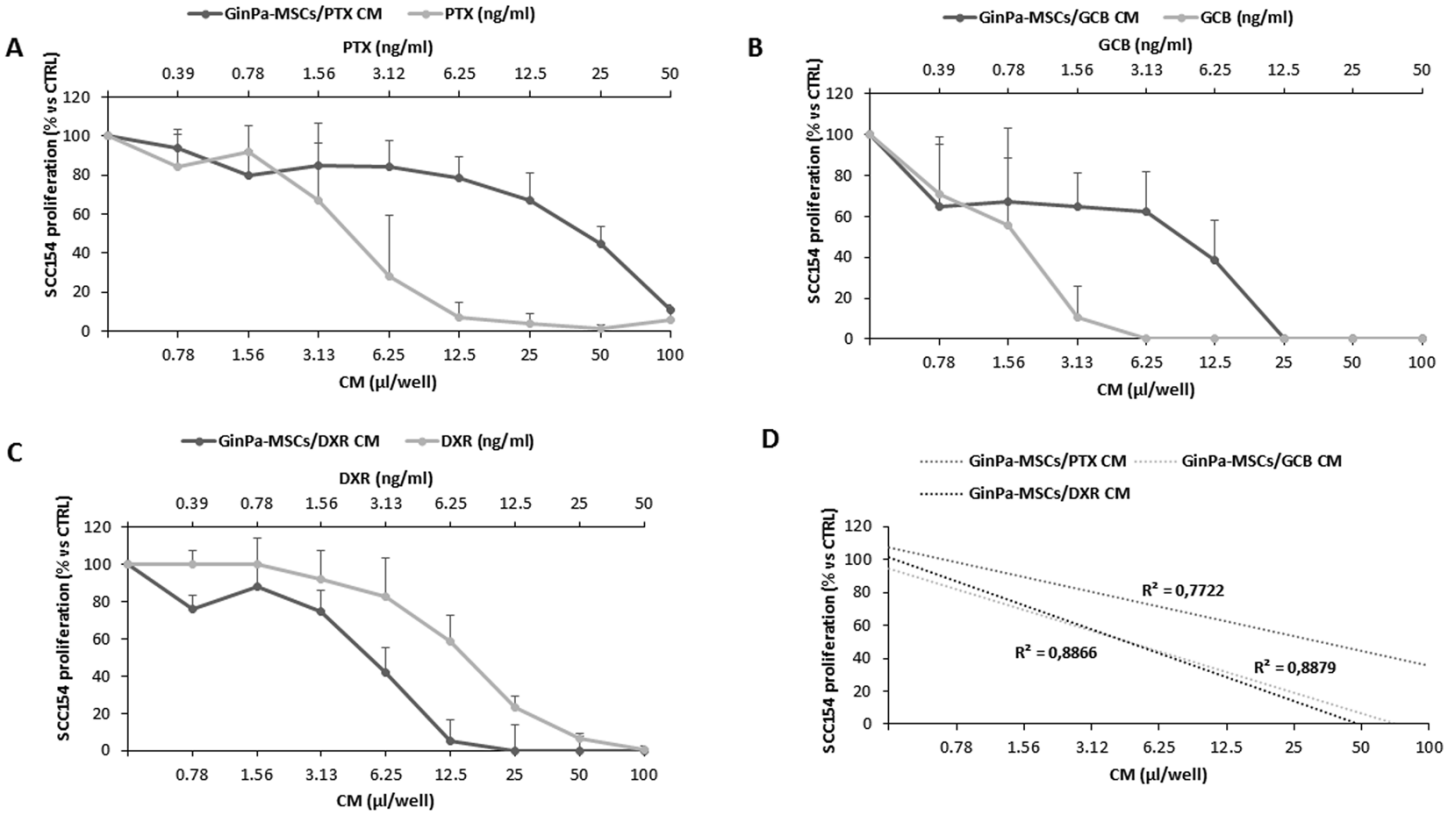
Anticancer activity against squamous cell carcinoma (SCC154) of conditioned medium (CM) from drug loaded GinPa-MSCs. The effect of conditioned media (CM) from GinPa-MSCs/PTX (**A**), GinPa-MSCs/GCB (**B**) and GinPa-MSCs/DXR (**C**), are expressed as percentage of the proliferation of SCC154 referred to that of untreated cells (control 100% proliferation). Values are expressed as mean ± standard deviation (SD) of three independent experiments. The regression and the correlation coefficient (R^2^) of the dose response kinetics are reported in figure (**D**).

The conditioned medium obtained from untreated GinPa-MSCs did not cause any modulation of the SCC154 proliferation (R^2^ = 0.004, p = 0.48) (Fig. 5A). The same conditioned medium was analysed in a microarray assay for 48 cytokines/growth factors and molecules detected at concentrations above 2 ng/ml are summarized in Fig. 5B. The GinPa-MSCs produced significant concentrations of some cytokines (Hu IL-6, Hu IL-8, Hu VEGF, Hu GROa, Hu SCGF-b; ranging from 13 to 24 ng/ml) but their presence in the secretoma do not seem to have some inhibiting or enhancing effect on SCC154 proliferation. On the contrary, the anti-tumor activity of the CM from GinPa-MSCs/PTX, GinPa-MSCs/GCB and GinPa-MSCs/DXR evaluated on SCC154 showed a dramatic inhibition of cancer cell growth. The study by linear regression of the dose response kinetics evidenced a significant coefficient of correlation (R^2^) ranging from 0.77 to 0.88 (Fig. 6).

## Discussion

The success of cancer chemotherapy consists of kill as many cancer cells as possible and producing the fewer possible toxic effects. With this intent, many strategies have been developed, including those based on new formulations of old anticancer compounds combined with new constructs as carriers such as nanoparticles, liposomes, proteins, etc. This strategy is based on the protection of the drug from degradation, the promotion of drug uptake by tumour mass as well as lower toxicity in healthy cells^22^. To improve the above strategies we evaluated the use of MSCs for the drug delivery^9, 13^ and we optimized the use of gingiva- derived MSCs (GinPa-MSCs) to uptake and release important and widely used anticancer agents DXR, GCB and PTX. For this purpose we applied to the SCC154 squamous carcinoma cell line the procedure we previously standardized for pancreatic carcinoma cells (19). In fact, although significant improvement in survival rate has been obtained in oral squamous carcinoma treatment, current therapies still cause severe toxicity that makes it necessary further research for more efficacious and safer new agents and treatments^23^.

In a first step, we compared the sensitivity of SCC154 to the three drugs according to a dose–response kinetics that evidenced the significant higher activity of GCB (Fig. 1). This result is in agreement with recent reports^24, 25^ suggesting gemcitabine as alternative agent to treat advanced squamous cell carcinoma. As expected, also the proliferation of GinPa-MSCs was dramatically affected by the three drugs and the highest activity was expressed by GCB (Fig. 2). The significant higher expression of hENT1 (the main carrier involved in gemcitabine transport in tumor cells)^21^ by GinPa-MSCs could explain the high sensitivity of GinPa-MSCs to GCB. (Figure 2D). The resistance of GinPa-MSCs to the cytocydal effect of GCB, DXR and PTX with, over 80% of living cells (Fig. 2D) enabled us to apply our standardized procedure of drug priming at very high concentration (2,000 ng/ml).

GinPa-MSCs were able to uptake and then release the three drugs in their conditioned medium. The anticancer activity was evaluated by a biological dosage assay against the carcinoma CFPAC-1 cells in both cell lysate and conditioned medium (Fig. 3). After 24 hour culture, drug-loaded GinPa-MSCs released approximately 60% of PTX internalized and approximately 90% and 100% of GCB and DXR, respectively (Fig. 4A). This probably reflects the different hydro-lipo-solubility of the dugs (eg: PTX is the most lipophilic and released in minor amount). Being GCB a pro-drug, this was an important confirmation that GCB was not inactivated by cell metabolism. Based on the evaluation of drug equivalent concentration (Fig. 4B), we estimated that each primed GinPa-MSCs could release 75 to 690 ng per 10^6^ cells (Fig. 4C). Furthermore, the CM of drug loaded Ginpa-MSCs exerted also a dramatic inhibition of the squamous carcinoma SCC154 proliferation.

Although the above results need to be confirmed by preclinical studies, they can be of help in predicting the drug concentration that could be possibly carried into the tumour environment *in vivo*. By applying the criteria adopted for cell therapy (i.e., 2 × 10^6^ MSCs/kg), we can estimate that the homing of such amount of MSCs to the tumour mass (or their *in situ* injection) can enable a local drug concentration at levels several times the IC_50_ values determined *in vitro*. In this context, to exclude a possible “collateral effect” resulting in a cancer stimulation exerted by GinPa-MSCs secretoma, we analysed the main cytokines/growth factors produced (Fig. 5B). The results confirmed that, in spite of high production of some factors (Fig. 5A), the secretoma of Ginpa-MSCs does not affect the *in vitro* growth of SCC154 whereas CM from drug primed GinPa-MSCs are very efficient in blocking tumor cell growth (Fig. 6).

The ability of GinPa-MSCs to uptake and release DXR allow us to discuss also some aspects concerning the toxicity that has been described for DXR new formulations (eg. pegylated liposomal doxorubicin, PLD). In fact, despite the significant decrease in cardiotoxic effects^26^ of these new DXR formulation, severe observations have been reported on their capacity to induce development of SCC as secondary malignancy. A secondary cancer may depend on many factors (e.g., the environment, food, pollutions etc.) and some cases are described that are drug treatment-related(eg: bladder cancer in cyclophosphamide or leukemia in alkylating agents treatment)^27^. However, some authors reported the cases of patients with ovarian cancer who developed squamous cell carcinomas after long treatment with PLD^28–30^; according to the authors, the long exposition to DXR released by PLD exerts to a toxic effect more significant than that exerted by free/non liposomal DXR, probably due to the specific accumulation of PDL into the oral mucosa. Although our data need to be further verified, they suggest that the treatment *in situ* of the ovary carcinoma by using DXR-loaded MSCs could avoid the mechanism of oral PLD concentration described.

As stated by Gisbon *et al*.^3^, the clinical management of squamous cell carcinomas can have benefit by using different approaches. One of them could be the drug delivery by MSCs, in particular from gingiva that according to Tomar *et al*.^31^, can be considered a better source of MSCs than the bone marrow. In addition, the collection of autologous GinPa-MSCs is an easy procedure that can be performed without any discomfort for the patient and it can be easily expanded to obtain large number of mesenchymal stromal cells also for banking. The use gingival MSCs as a carrier for delivery anti-tumor molecules is of great interest also for the anatomic homology of the source with the type of cancer originating into the oral cavity. This means that drug loaded GinPa-MSCs with anticancer agents, GCB or PTX in particular, could be suitable for possible *in situ* advanced cell therapies (e.g., in pre-cancerous stage, as adjuvant therapy to reduce the toxicity related to systemic treatment, to prevent cancer recurrences after surgical treatment, to reduce the radiation treatment effects, etc.
